# Editorial: How best to deliver disability care in a cost-effective way: improving health care delivery and health outcomes for people with disability

**DOI:** 10.3389/fresc.2023.1307810

**Published:** 2023-10-30

**Authors:** Corneliu Bolbocean

**Affiliations:** Nuffield Department of Primary Health Sciences, University of Oxford, Oxford, United Kingdom

**Keywords:** rehabilitation, people with disabilities (PD), quality of life outcomes, cost-effectiveness (CE), service delivery, vulnerable groups

**Editorial on the Research Topic** How best to deliver disability care in a cost-effective way: improving health care delivery and health outcomes for people with disability

The International Classification of Functioning, Disability, and Health (ICF) defines “disability” as a holistic term encompassing impairments, limitations in activities, and restrictions in participation (1). Individuals with disabilities tend to have suboptimal health outcomes compared to those without disabilities for multiple reasons (2). These individuals might also face a higher risk of secondary health issues, such as negative mental health outcomes (3) as well as face high risks of adverse health and socioeconomic outcomes (4, 5).

Rehabilitation is defined as a comprehensive set of interventions aimed at addressing impairments, limitations in activities, and restrictions in participation (1). It also considers personal and environmental factors that influence functioning. The goal of rehabilitation is to enhance the overall functioning of individuals with disabilities. The overall evidence regarding the effectiveness and cost-effectiveness of rehabilitation services is sparse. This could partly account for the historically minimal attention rehabilitation has received from governments which resulted in limited service availability in numerous countries (6).

This Research Topic, entitled “How Best to Deliver Disability Care in a Cost-Effective Way: Improving Health Care Delivery and Health Outcomes for People with Disability”, contains five novel and important studies that shed new light on disability-related healthcare (Hale et al., Novak et al., Limacher et al., Verpe et al., Shibata et al.). The central goal of this Research Topic was to promote research on health economics related to disability, facilitating an informed decision-making regarding rehabilitation interventions. Their unique, yet interconnected focus areas address several important gaps in the literature and overall underscores the importance of a comprehensive approach to health provision given the changing technological landscape. Furthermore, it underlines the need to account for tailored care, socioeconomic circumstances, and modern technology in devising effective healthcare strategies for individuals living with disabilities.

The first study by Hale et al. focuses on the Diabetes Community Exercise Programme and uncovers the significance of a patient-oriented approach in inspiring adults with type 2 diabetes to participate in regular physical activities (Hale et al.). The research illustrates the importance of observation, personalised workout plans, adaptable educational components, and well-trained healthcare professionals in the success of this model. Nevertheless, the study also emphasizes that an effective healthcare delivery system must not ignore the challenges linked to logistics and administration. Authors advocated for a balanced and holistic healthcare delivery approach.

Measuring patient-reported outcomes such as quality of life outcomes is essential for health economic evaluation related to disability interventions (Shibata et al.). Shibata et al. present a novel application of speech analytics and machine learning to estimate quality of life outcomes (QoL) in schizophrenia. By utilizing machine learning models to estimate patients' QoL based on their speech patterns, the authors demonstrate the possibility for continuous monitoring. Automated monitoring could make patient-centered care more scalable. However, economic modeling is required to compare costs and outcomes of this novel technology-assisted approach relative to standard psychiatric care. Furthermore, similar methods may be adaptable to other disabilities.

The third study by Limacher et al. assesses the relationship between socioeconomic status (SES), gender, and health outcomes among physically challenged individuals in Morocco (Limacher et al.). The study shows that perceived SES indicators, such as economic hardship and lower societal standing, have a substantial impact on health outcomes. The study highlights the complexity of the relationships between socioeconomic gradient of health and health outcomes; hence highlighting the need for more detailed investigations to fully understanding this relationship. This research also underscores the need to address social determinants of health in order to mitigate the dual burden of disability and social disadvantages, especially, in settings with limited resources within the developing countries. Policymakers can use these findings to formulate interventions aimed at easing social marginalization and poverty, and foster more equitable health outcomes.

The structured model of Novak et al. for evidence-based rehabilitation decisions emphasizes the comprehensive assessment and personalized interventions (Novak et al.). Explicit incorporation of prognosis conversations and client preferences displays person-centered principles. Testing the model's impact on service delivery costs, patient satisfaction, and functional gains would further demonstrate the value of the model. The team-based adaptations could be beneficial given multi-professional collaboration in disability care.

The comparative evaluation of instruments which seemingly measure the same constructs is relatively well documented within the quality of life research literature (7–10). However, the comparison of quality of life measures with objective tests is less common in the literature. The study by Verpe et al. examined the construct validity of the Danish version of the Work Rehabilitation Questionnaire (WORQ) by comparing its physical capacity items to objective physical capacity tests and selected SF-36 physical function items in 40 job center clients (Verpe et al.). Moderate to strong correlations were found between WORQ and SF-36 items, and weak to moderate correlations between WORQ physical items and the following physical capacity objective tests: 30-s sit-to-stand-test, a handgrip-strength test, and a 6-min walk test to estimate cardiorespiratory fitness. Calculations showed higher negative predictive values than positive predictive values overall, with varying sensitivity and specificity. However, Verpe et al. were able to provide evidence for the construct validity of WORQ-Danish, but also raise questions about whether objective tests are the gold standard for functional assessment. Furthermore, the authors suggest more research on WORQ's screening capabilities alongside other self-report and objective measures to provide complementary information for guiding work-related actions.

Overall this Research Topic demonstrates that patient-centered care requires paying close attention to individual needs and preferences while also leveraging expertise, data, and technology. Fruitful research directions should focus on generating rigorous evidence to guide disability care, while keeping the perspectives of people with disabilities at the center. Recent high-tech solutions to optimize rehabilitation interventions warrant further exploration and rigorous health economic evaluations.

## References

[1] World Health Organization. Towards a common language for functioning, disability, and health: icf. In: The international classification of functioning, disability and health. Geneva: World Health Organization (2002).

[2] Bethge M Von Groote P Giustini A Gutenbrunner C. The world report on disability: a challenge for rehabilitation medicine. Am J Phys Med Rehabil. (2014) 93(1):S4–11. 10.1097/PHM.0000000000000016 24356081

[3] Prince M Patel V Saxena S Maj M Maselko J Phillips MR No health without mental health. Lancet. (2007) 370(9590):859–77. 10.1016/S0140-6736(07)61238-0 17804063

[4] Banks LM Kuper H Polack S. Poverty and disability in low-and middle-income countries: a systematic review. PLoS One. (2017) 12(12):e0189996. 10.1371/journal.pone.0189996 29267388 PMC5739437

[5] Smythe T Zuurmond M Tann CJ Gladstone M Kuper H. Early intervention for children with developmental disabilities in low and middle-income countries– the case for action. Int Health. (2021) 13(3):222–31. 10.1093/inthealth/ihaa044 32780826 PMC8079317

[6] Bright T Wallace S Kuper H. A systematic review of access to rehabilitation for people with disabilities in low-and middle-income countries. Int J Environ Res Public Health. (2018) 15(10):2165. 10.3390/ijerph15102165 30279358 PMC6210163

[7] Harrison MJ Davies LM Bansback NJ McCoy MJ Verstappen SMM Watson K The comparative responsiveness of the EQ-5D and SF-6D to change in patients with inflammatory arthritis. Qual Life Res. (2009) 18(9):1195–205. 10.1007/s11136-009-9539-2 19777373 PMC2761817

[8] Richardson J Khan MA Iezzi A Maxwell A. Comparing and explaining differences in the magnitude, content, and sensitivity of utilities predicted by the EQ-5D, SF-6D, HUI 3, 15D, QWB, and AQoL-8D multiattribute utility instruments. Med Decis Making. (2015) 35(3):276–91. 10.1177/0272989X14543107 25159172

[9] Kennedy-Martin M Slaap B Herdman M van Reenen M Kennedy-Martin T Greiner W Which multi-attribute utility instruments are recommended for use in cost-utility analysis? A review of national health technology assessment (HTA) guidelines. Eur J Health Econ. (2020) 21(8):1245–57. 10.1007/s10198-020-01195-8 32514643 PMC7561556

[10] Bolbocean C Anderson PJ Bartmann P Cheong JL Doyle LW Wolke D Comparative evaluation of the health utilities index mark 3 and the short form 6D: evidence from an individual participant data meta-analysis of very preterm and very low birthweight adults. Qual Life Res. (2023) 32:1703–16. 10.1007/s11136-023-03344-x 36705795 PMC10172285

